# Eye Nutrition in Context: Mechanisms, Implementation, and Future Directions

**DOI:** 10.3390/nu5072483

**Published:** 2013-07-05

**Authors:** Barbara Demmig-Adams, Robert B. Adams

**Affiliations:** Department of Ecology & Evolutionary Biology, University of Colorado, Boulder, CO 80309-0334, USA; E-Mail: Robert.Adams@colorado.edu

**Keywords:** antioxidants, carotenoids, gene regulation, light collection, lipid peroxidation, lutein, photo-damage, photoprotection, programmed cell death, zeaxanthin

## Abstract

Carotenoid-based visual cues and roles of carotenoids in human vision are reviewed, with an emphasis on protection by zeaxanthin and lutein against vision loss, and dietary sources of zeaxanthin and lutein are summarized. In addition, attention is given to synergistic interactions of zeaxanthin and lutein with other dietary factors affecting human vision (such as antioxidant vitamins, phenolics, and poly-unsaturated fatty acids) and the emerging mechanisms of these interactions. Emphasis is given to lipid oxidation products serving as messengers with functions in gene regulation. Lastly, the photo-physics of light collection and photoprotection in photosynthesis and vision are compared and their common principles identified as possible targets of future research.

## 1. Introduction

Carotenoids possess functions as diverse as their colors (for a review, see [1]). Carotenoid function as visual cues is matched by roles in (1) visual detection of these cues and (2) protection of the vision process against overly bright light. Many animals are able to detect visual signals carrying important information content about their environment via the light-absorbing rhodopsin, formed from the carotenoid (β-carotene) cleavage product vitamin A combined with the opsin protein. On the other hand, the carotenoids zeaxanthin and lutein provide essential protection of the eye in humans and many other animals. Moreover, carotenoids are involved in the regulation of life-and-death processes at the cellular level via modulation of signaling networks that control cell division and programmed cell death [2]. The present overview places carotenoids into the context of (1) familiar organisms and objects colored by carotenoids, (2) their roles in human vision, with an emphasis on protection by zeaxanthin and lutein against vision loss, (3) various dietary sources of zeaxanthin and lutein, (4) synergistic interactions of zeaxanthin and lutein with other dietary factors affecting human vision and the emerging mechanisms of these interactions, and (5) a comparison of the photo-physics of light collection and photoprotection in photosynthesis and vision and their common principles that might be rewarding targets of future research.

## 2. Colors and Nomenclature of Carotenoids

The flamingo’s pink plumage, the yellow of egg yolks and daffodils, the orange of peppers, and yellow-orange autumn foliage are colors conferred by carotenoids (see [1]). It is plants and photosynthetic microbes that synthesize most carotenoids; non-photosynthetic consumers, like humans and other animals, rely strictly on acquiring essential carotenoids with their food and are unable to synthesize these *de novo* (although some conversions are known to occur).

Carotenoids are typically named after organisms in which they occur in high concentrations and/or from which they were first isolated and identified. For example, *carot*ene colors the carrot and *zea*xanthin levels are high in the corn plant (scientific name: *Zea mays*). The names of the two major groups of carotenoids, carotenes (carotenoids without oxygen atoms in the molecular structure) and xanthophylls (carotenoids containing oxygen atoms) further relate to their features: -*ene* (as in carot*ene*) for the presence of many carbon-carbon double bonds (from the chemical nomenclature of -*ene* for molecules with carbon-carbon double bonds) and *xantho-phyll* (Greek for “yellow-leaf”) in reference to the high level of these carotenoids in yellow autumn leaves. Zeaxanthin has a structural isomer, lutein; even though these two xanthophylls differ merely in the placement of a single C=C double bond, they possess discernible biological functions.

## 3. Carotenoids in the Functioning and Protection of the Human Eye

The dietary carotenoid β-carotene is provitamin A that, after being cleaved, yields two molecules of vitamin A as the chromophore (light-absorbing) component of rhodopsin. In addition, vitamin A serves as a modulator of genes serving in the immune response [1]. Chronic severe vitamin A deficiency therefore causes not only blindness, but also often death from infectious disease.

In addition to serving as precursors of constituents of the human eye, carotenoids are thought to protect the vision process, improve visual acuity and shape discrimination, and be involved in the prevention of cataracts and age-related blindness (age-related macular degeneration or AMD) (for reviews, see [3,4]). Rather than carotenes, it is zeaxanthin and lutein—two carotene-derived xanthophylls synthesized by plants and algae—that are chiefly involved in protection of the vision process.

Dietary zeaxanthin and lutein—neither of which, as stated above, can be synthesized by humans—apparently confer *multiple* beneficial effects to human health. Epidemiological studies have identified strong inverse trends between zeaxanthin and/or lutein consumption and human diseases, including age-related eye disease, various cancers, and other conditions [5,6,7]. The underlying mechanisms for these protective effects have yet to be fully elucidated (see Section 5 below). Plants and photosynthetic microbes synthesize zeaxanthin and lutein for their own protection against damage by intense sunlight—and the same two xanthophylls, when consumed with the human diet, apparently also protect the human eye from damage by intense light [5].

In the human eye, zeaxanthin and lutein (as well as some meso-zeaxanthin, formed from lutein) are the predominant carotenoids in the yellow spot (*macula lutea*) of the retinal macula, with higher ratios of zeaxanthin to lutein in those areas receiving the highest light exposure [8,9]. Both xanthophylls are accumulated in the blood stream (from the intestinal tract), and then further accumulated in the retina (from the blood stream). Along with this overall accumulation of xanthophylls in the eye, the ratio of zeaxanthin to lutein increases at each step (from the intestinal tract to the blood plasma to the eye), indicating a particular importance of zeaxanthin in the retina (for reviews, see [3,4,6]). Individuals suffering from age-related eye disease (such as AMD) possess lesser xanthophyll densities throughout their retinas, and there is an inverse correlation between dietary zeaxanthin and lutein intake and the risk for AMD as well as cataracts [6].

It has been suggested that the yellow zeaxanthin and lutein pigments act by providing an absorbing shield against the most harmful blue portion of sunlight and perhaps also against UV light [10], but evidence for additional or alternative functions is mounting [2,4,10]. Zeaxanthin and lutein act synergistically with the antioxidant vitamin E (and likely other antixodants), as well as with certain (omega-3) poly-unsaturated fatty acids, in photoreceptor protection (for details, see Section 4 below).

A breakthrough in the understanding of the function of retinal zeaxanthin in protecting the eye was made by the discovery that dietary zeaxanthin *prevents* programmed cell death of retinal photoreceptor cells in an intact animal model [11,12]. Because of the latter finding, one might wonder whether dietary zeaxanthin would increase cancer risk by inhibiting programmed cell death of cancer cells. However, this concern is unfounded since dietary zeaxanthin has, in fact, been associated with a lower cancer risk (see, e.g., [13]). Consumption of dietary zeaxanthin is therefore not only correlated with improved eye health but also with a lower cancer risk. While the mechanism of cancer prevention by carotenoids is presently unknown, it may involve an actual *stimulation* of programmed cell death of various cancer cells [14,15,16], including cancer of the eye [17]. The xanthophylls zeaxanthin and lutein share this remarkable ability, *i.e.*, to simultaneously *protect needed* cells and apparently *destroy unwanted* cells, with several other classes of dietary compounds like some phenolics and omega-3 fatty acids ([2]; see also Section 5 below). In addition to their protective effects against vision loss, zeaxanthin and lutein apparently also serve in improving vision overall. Consistent with its preferential concentration in the central region of the retina (in the fovea), a dietary supplement of zeaxanthin (8 mg daily) specifically enhanced high-contrast visual acuity and shape discrimination, while a dietary supplement of lutein (9 mg daily), consistent with its preferential distribution in the non-central regions of the retina, enhanced low-contrast visual acuity and glare recovery [3].

While zeaxanthin and lutein levels in the human retina are correlated with dietary intake of these xanthophylls, genetic factors also play a role [4,18]. Individuals with a darker iris color (with greater levels of melanin pigment) possess higher retinal levels of zeaxanthin and lutein [19]. It will be important to assess whether these differences represent a genetic difference in the ability to enrich zeaxanthin and/or lutein from the diet among individuals and populations, or whether a darker iris may prevent xanthophyll destruction by intense light.

## 4. Dietary Sources of Zeaxanthin & Lutein

There is evidence that the human consumer should avoid excessive supplementation with carotenoids [20,21]. For example, daily supplementation with excessive amounts of β-carotene for several years actually increased the risk of Finnish male smokers for lung cancer. In addition, blue-green algal (cyanobacterial) supplements (with high levels of a class of highly oxygenated xanthophylls called ketocarotenoids) caused crystalline ketocarotenoid deposits in the human eye [22]. Currently available blue-green algal supplements thus need to be viewed with caution, due to potential adverse effects of ketocarotenoid accumulation.

### 4.1. Carotenoid and Vitamin Supplements

There are additional reasons why the human consumer should avoid high-dose supplements. A comprehensive review [21] of a large number of clinical studies examining the effect of antioxidant and/or carotenoid supplements on chronic diseases demonstrated opposite effects dependent on dosage. Supplements doses similar to, or up to a few times higher than, the US daily recommended allowance (RDA) typically had positive effects (reducing disease risk), while higher doses had no effect on disease risk, and extremely high doses, above ten times the RDA, had negative effects (increasing disease risk) [21]. Some studies have reported benefits of lutein and zeaxanthin supplements (10–20 mg daily; [23,24]) for AMD patients, while other studies reported no benefits of 6 mg lutein combined with vitamins and minerals [25]. More research is needed to further explore effects of different doses of supplementation for patients already diagnosed with eye disease.

On the other hand, prevention of eye disease prior to onset may be well served by regular consumption of xanthophylls and other dietary factors as part of a whole diet based on foods containing multiple, synergistically acting ingredients. The underlying mechanisms why whole foods rich in, e.g., antioxidants and carotenoids protect against disease, while high-dose antioxidant supplements can have the opposite effects, are currently not fully understood. However, it appears that reactive oxygen species and cellular signaling networks are involved. Reactive oxygen species, which are removed by antioxidants and carotenoids, actually exert essential positive effects at low levels (they are the signals triggering up-regulation of the body’s own internal antioxidant defenses), while being able to cause tissue damage at unchecked high levels (see, e.g., [26,27,28]). Section 5 below addresses cellular signaling networks that may be involved.

### 4.2. Yellow Foods

Eggs, yellow corn, and yellow peppers are probably the richest dietary sources of zeaxanthin and lutein in the United States. These food sources receive all of their yellow-to-orange color from their zeaxanthin and lutein content. In contrast, green leafy plant foods typically contain high levels of lutein, but very little zeaxanthin. This difference is due to the fact that green leaves form zeaxanthin under full sunlight and remove zeaxanthin when no longer exposed to full sunlight (for additional details, see section on “leafy greens and other plant sources” below). In contrast to green leaves, certain non-photosynthetic parts of plants, like the ear of corn, accumulate *constant high* levels of zeaxanthin. Yellow corn is a food naturally high in zeaxanthin and lutein, and all of its yellow color stems directly from these two pigments (see [29] for analysis of various corn products). White corn, on the other hand, does not provide these xanthophylls. Lines of corn with even higher levels of zeaxanthin and lutein might be identified from locally adapted varieties, or could be produced by further breeding and/or engineering.

Chicken eggs are another food with high levels of both zeaxanthin and lutein. However, chickens are no more capable of synthesizing their own xanthophylls than humans, and the coloration (and zeaxanthin and lutein content) of their eggs strictly depends on the chickens’ feed. As long as a xanthophyll-rich food source is available (from alfalfa, corn, or other sources containing zeaxanthin and lutein), chickens deposit zeaxanthin and lutein into their eggs; and egg color varies with carotenoid content. Furthermore, just as different strains of corn have different levels of zeaxanthin and lutein, some chicken breeds deposit more zeaxanthin and lutein into their eggs than others [30].

### 4.3. Leafy Greens and Other Plant Sources

Just as is the case for animals, plants and algae also use carotenoids for *both light collection AND photoprotection* against the destructive effects of intense light. The xanthophylls zeaxanthin and lutein stand out as primary agents of photoprotection in plants. Zeaxanthin facilitates the safe removal of potentially damaging excessive excitation energy [5,31,32]; zeaxanthin’s close isomer lutein plays a minor role in the same process of the dissipation of excessive excitation [33]. In addition, zeaxanthin also provides plant photoprotection by direct inhibition of the oxidation of lipids of the photosynthetic membrane (lipid peroxidation; [34,35]; see also Section 5 below).

How much zeaxanthin versus lutein can be obtained from leafy green plant foods varies greatly, as stated above. While the green parts of plants after harvest typically contain high levels of lutein, they retain mere traces of zeaxanthin. This is because plants carefully modulate the level of zeaxanthin in response to the amount of light they absorb. Leaves only produce zeaxanthin (via de-epoxidation of another xanthophyll, violaxanthin, that consists of a zeaxanthin molecule with two epoxide groups added) when exposed to high light; whenever direct high light exposure ends, zeaxanthin is quickly re-converted (re-epoxidized) to its direct xanthophyll precursor (violaxanthin). This fine control of zeaxanthin levels is important for the plant to (1) maintain its ability to safely dissipate potentially destructive excessive light one minute (via zeaxanthin as a dissipater of excess light) and (2) to quickly return to efficiently using sunlight for sugar production the next, by converting the dissipater zeaxanthin back to its direct, non-dissipating xanthophyll precursor [31,36,37].

Human consumption of zeaxanthin is highly desirable because zeaxanthin needs to be preferentially accumulated and incorporated into the parts of the mammalian retina exposed to high irradiance ([8]; see also above). For this reason, an arrest of the back-conversion of zeaxanthin to its precursor in green leaves may be a desirable trait to incorporate into crops that provide green leafy foods. Such a retention of zeaxanthin can be accomplished, e.g., by knocking out or silencing the enzyme/gene (zeaxanthin epoxidase) responsible for zeaxanthin conversion to its xanthophyll precursor and/or by overexpressing enzymes in earlier portions of the carotenoid biosynthetic pathway. Over-expression of the enzyme catalyzing synthesis of zeaxanthin from β-carotene in the plant model species *Arabidopsis* led to elevated zeaxanthin accumulation [38].

Additional mutants unable to convert zeaxanthin to its xanthophyll precursor, and thus accumulating high levels of zeaxanthin, have been produced in model plants and algae [39] and these traits can be transferred to crop plants. However, since constantly elevated zeaxanthin levels may diminish the green leaf’s ability to efficiently collect sunlight during the parts of the day when light levels are low and limiting to photosynthesis, e.g., early morning and late afternoon, the effect of continuous zeaxanthin retention in leaves on the productivity of crop plants needs to be further examined. Due to the need of leaves to photosynthesize efficiently for maximal biomass production, overexpression of zeaxanthin in fruit, rather than leaves, is attractive. Tomato fruit with increased zeaxanthin content has recently been engineered via two manipulations (overexpression of lycopene-cyclase and of β-carotene hydroxylase; [40]). Zeaxanthin-rich potato has also been produced [41] as well as zeaxanthin-accumulating *E. coli* [42]. While foods engineered to contain high zeaxanthin levels should aid in augmenting dietary zeaxanthin supply for human populations at risk for zeaxanthin deficiency, attention should be given to avoiding excessive consumption of such fortified foods.

## 5. Xanthophylls in the Context of Other Dietary Modulators

Reviews of lifestyle- and diet-associated risk factors for age-related macular degeneration indicate that, in addition to zeaxanthin and lutein, omega-3 fatty acids, antioxidant vitamins C and E, antioxidant minerals, and other dietary factors lower the risk for AMD [43]. These factors act synergistically (via the emerging mechanisms reviewed below) in modulating key signaling networks.

### 5.1. Foods Contain Multiple Gene Regulators Acting in Synergy

The essential role of food components—such as vitamins—in human physiology has long been recognized. However, it is only now being realized that a multitude of additional dietary factors have profound effects on human health and the risk for disease, and may thus deserve vitamin status as well (see [5]). Many of these dietary factors possess the remarkable ability to alter expression of regulatory genes that modulate human metabolism. Most of these dietary factors are synthesized by plants or algae and are referred to as phytochemicals (from *phyto* = plant), phytonutrients, or nutraceuticals. These dietary factors modulate expression of central human genes (master control genes) that regulate processes of fundamental importance, such as cell proliferation, programmed cell death, and the immune response (see, e.g., [5,44,45]). These key processes, and any imbalances in their regulation, play a major role in all major human diseases including cancer, autoimmune diseases, and pro-inflammatory diseases (such as age-related blindness, heart disease, diabetes, and others; [46]). Macular degeneration involves excessive programmed cell death as well as inflammation. While the original literature on programmed cell death distinguishes between “programmed cell death” and “apoptosis” as a particular form of cell death, both of these processes are referred to as programmed cell death in the present review for simplicity’s sake.

Dietary factors may be able to *stop* programmed cell death of vital cells and *promote* programmed cell death of unwanted cells. Such a potential to both *trigger programmed cell death of unwanted cells* while *aiding in the survival of needed cells* is not only demonstrated by zeaxanthin and lutein, but also by poly-unsaturated omega-3 fatty acids (in fish that consume omega-3 fat-producing cold-water algae; [47]), and plant compounds (phenolics) with multiple functional (phenol) groups conferring exceptionally strong antioxidant qualities to the molecule [44,48]. This potential makes these food-derived compounds highly desirable nutraceuticals.

Studies with human cancer cell lines indicate that lutein is able to induce programmed cell death of human breast cancer cells [14,15] and leukemia cells [16]. Similarly, zeaxanthin is able to promote programmed cell death of eye cancer (neuroblastoma) cells [17]. Lutein furthermore induced programmed cell death in mouse tumor cells, but decreased programmed cell death in cancer-fighting immune cells of tumor-bearing mice [14].

### 5.2. Gene Regulators Produced by Lipid Peroxidation

**Figure 1:**
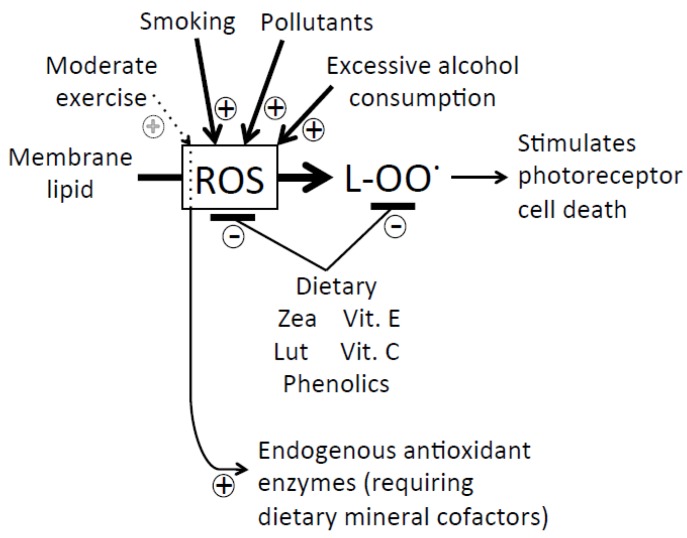
Schematic depiction of (1) the oxidation (by reactive oxygen species, ROS) of membrane lipids to lipid peroxides (l-OO^•^) that are further converted to messengers, e.g., stimulating photoreceptor cell death as well as (2) the effect of lifestyle/environmental/dietary factors on ROS and/or lipid peroxide levels. Moderate exercise generates moderate amounts of ROS serving to trigger full formation of endogenous antioxidant defenses (in the form of antioxidant enzymes requiring dietary minerals as their cofactors). Smoking, exposure to pollutants, and excessive alcohol consumption all strongly increase ROS levels. Dietary zeaxanthin (Zea), lutein (Lut), vitamins E (Vit. E) and C (Vit. C), and phenolics, as well as endogenous antioxidant enzymes, serve in synergy to lower the levels of ROS (via ROS detoxification) as well as recycle lipid peroxides (via re-reduction). Stimulation by ROS, and inhibition by various antioxidants, applies equally to non-enzymatic and enzymatic (via lipoxygenase, LOX) lipid peroxidation. Lipoxygenases contain a catalytic iron center that is active in lipid peroxidation only after oxidation (by ROS) to (active) LOX-Fe^3+^ [53], and can be inactivated by antioxidants to (inactive) LOX-Fe^2+^ [54]. Products of both enzymatic and non-enzymatic lipid peroxidation serve as gene regulators.

In both plants and animals, membrane lipids are actively oxidized by enzymes (e.g., lipoxygenases) that function as part of cellular signaling networks and actively respond to oxidative challenges. Lipoxygenases produce messengers that regulate programmed cell death, the immune response, and other vital processes. Lipoxygenases in both plants and animals produce multiple lipid-derived messengers (in plants collectively termed “oxylipins”, such as jasmonic acid, and in animals collectedly termed “eicosanoids”, such as prostaglandins, leukotrienes, and thromboxanes). Each messenger family typically includes members with *opposite* regulatory functions, *i.e.*, that either *promote* or *inhibit* programmed cell death ([49,50]; see Figure 1). For example, *inhibition* of lipoxygenases *prevented programmed cell death of neurons* and was discussed as a new target for the treatment of Alzheimer’s disease [51], whereas *inhibition* of lipoxygenases in leukemia cells *actively induced programmed cell death* [52]. As further detailed in the legend of Figure 1, lipoxygenases are activated by oxidants (e.g., reactive oxygen species, ROS) and are inhibited by various antioxidants. Reactive oxygen species thus activate both enzymatic and non-specific, non-enzymatic lipid peroxidation, and multiple products of both enzymatic and non-enzymatic lipid peroxidation serve as gene regulators. Likewise, antioxidants inhibit both enzymatic and non-enzymatic lipid peroxidation.

While it had been known for some time that the lipid-soluble vitamin E is able to suppress (both non-enzymatic and enzymatic) lipid peroxidation, it is now emerging that zeaxanthin and other dietary factors interact synergistically with vitamin E (see below). Suppression of lipid-peroxidation-derived modulators of programmed cell death (and/or other vital cellular processes) is an attractive mechanism for these dietary modulators’ ability to protect vital cells, while eliminating unwanted cells. This fine-tuned modulation of vital metabolic functions emphasizes the critical importance of a *balanced* dose of these dietary regulators.

Experimentation with isolated biological membranes showed that lipid-soluble antioxidants (like zeaxanthin and vitamin E) synergistically protect against oxidation of unsaturated fatty acids (that are highly susceptible to oxidation; see Figure 1). While massive oxidation of membrane lipids eventually leads to membrane damage, a more physiologically relevant effect is the rapid formation of small amounts of oxidized lipid derivatives serving as messengers that modulate the expression of genes involved in programmed cell death and other vital functions (Figure 1).

Zeaxanthin *inhibits* the oxidation of membrane lipids (lipid peroxidation; Figure 1) in plants [34,35] as well as in humans (e.g., in epithelial cells of the eye’s lens; [55]). Zeaxanthin also protects lipids against peroxidation *in vitro*, and zeaxanthin’s ability to provide this protection is enhanced by addition of vitamin E, with zeaxanthin having the more potent, primary effect [56,57]. While zeaxanthin and vitamin E act synergistically to suppress lipid peroxidation in biological membranes, vitamin C aids in the recycling of vitamin E at the membrane/cytosol interface, and various phenolics (with strong antioxidant effects) can do the same. Furthermore, antioxidant minerals, such as zinc, copper, and selenium, are essential cofactors of the body’s internal antioxidant enzymes that also serve in the cellular antioxidant network (Figure 1). Others have drawn similar conclusions and recommended supplements consisting of cocktails of the factors above (specifically, vitamins A, B, C, E, β-carotene, zeaxanthin, lutein, selenium, zinc, and the herb *Gingko biloba*; [58]).

Dietary antioxidants must work in conjunction with the body’s own internal antioxidant enzymes (Figure 1). Antioxidant enzymes require mineral cofactors from the diet, and interact with dietary xanthophylls, vitamins, and phenolics to suppress unchecked accumulation of reactive oxygen species. However, it is small amounts of reactive oxygen species (produced, e.g., via moderate physical exercise) that serve as the vitally important trigger for synthesis of the body’s own antioxidant enzymes (Figure 1; [27]). It was recently been proposed that, “reactive oxygen species (ROS) and chronic oxidative changes in membrane lipids and proteins found in many chronic diseases are not the result of accidental damage. Instead, these changes are the result of a highly evolved, stereotyped, and protein-catalyzed ‘oxidative shielding’ response that all eukaryotes adopt when placed in a chemically or microbially hostile environment” [28]. The requirement of small amounts of ROS for induction of endogenous antioxidant defenses is the likely reason for the adverse effects of high-dose antioxidant supplements that presumably abolish the beneficial effects of ROS [21].

### 5.3. Poly-Unsaturated Fatty Acids

Exposure to reactive oxygen (readily formed in highly oxygenated tissues, such as the human eye) leads to preferential peroxidation of poly-unsaturated fatty acids. Poly-unsaturated fatty acids are linked to eye disease (as well as multiple other chronic diseases) in both positive and negative ways. Dietary poly-unsaturated fatty acids fall into two major groups, *i.e.*, omega-6 (mainly linoleic acid and arachidonic acid) and omega-3 fatty acids, mainly alpha-linolenic acid, *e*icosa*p*entaenoic *a*cid (EPA) and *d*ocosa*h*exaenoic *a*cid (DHA). A balanced ratio of dietary omega-6 to omega-3 fats (below 10:1 and perhaps as low as 2:1) is needed to support human health (including the health of the human eye), while the modern western diet provides a highly unbalanced ratio of about 10–20:1 [59]. Such a high, unbalanced ratio of omega-6 to omega-3 fatty acids is thought to promote programmed cell death and inflammation in some tissues, thereby increasing the risk of (1) chronic pro-inflammatory disease, like eye disease [43], diabetes, and heart disease [60,61], and a host of neuropsychiatric and neurodevelopmental disorders ([62,63]; see also [64]), as well as promoting (2) excessive cell proliferation (cancer) of other tissues [65,66].

How do poly-unsaturated fatty acids interact with vital cellular signaling networks? Both groups of fatty acids are essential (they cannot be synthesized in the human body), and form the precursors of lipid peroxidation-based regulators of gene expression discussed above. Both omega-6 and omega-3 poly-unsaturated fatty acids become incorporated into membrane lipids, where they represent the lipids most highly susceptible to either non-enzymatic or enzymatic oxidation (the unsaturated bonds of fatty acids are easily oxidized).

Oxidatively modified derivatives of omega-6 and omega-3 fatty acids typically serve as antagonists of each other in the regulation of gene expression, such that a balanced, relatively low ratio of omega-6 to omega-3 is required to prevent programmed cell death, e.g., in the eye. The omega-3 fatty acid DHA is concentrated in photoreceptor cells, and a major recent review concluded that, “DHA is necessary for vision, photoprotection, and corneal nerve regeneration” [67]. Furthermore, omega-3 supplementation has been demonstrated to prevent programmed cell death of tear gland cells [68]. Preferential lutein and zeaxanthin accumulation in membrane domains rich in poly-unsaturated fatty acids has been suggested to prevent lipid peroxidation [69]. Figure 1 focuses on stimulation by ROS, and inhibition by antioxidants, of the production of photoreceptor-death-stimulating messengers presumably derived from omega-6 fatty acids. Future research is needed to fully elucidate the effects of messengers derived from omega-3 fatty acids, and what relative and absolute concentrations of omega-6 and omega-3-derived messengers are needed to protect photoreceptors and other cells.

Current dietary recommendations are to keep the sum of all poly-unsaturated fats consumed in the human diet between 15% and 20%–30% of total fat consumption, with most of the remainder coming from mono-unsaturated fats like oleic acid found in many plant foods. While most currently used sunflower, corn, soybean, or cottonseed oils are excessively high in omega-6 fatty acids, utilization of high-oleic varieties of these oil-seed crops that either naturally contain, and/or have been genetically engineered to contain, much reduced levels of omega-6 linoleic acid should have many health benefits [70,71,72,73,74].

### 5.4. Dietary and Lifestyle Factors Increasing Disease Risk

High levels of saturated fats and/or trans fats and a high glycemic load (high and frequent consumption of carbohydrates that rapidly break down into simple sugars) as well as smoking, excess alcohol consumption, and physical inactivity promote the production of pro-inflammatory messengers and/or unchecked high levels of reactive oxygen species, and increase the risk for chronic, pro-inflammatory disease (see, e.g., [75]). Again, regular exercise (and consumption of the required mineral cofactors) is required to fully induce the body’s own internal complement of antioxidant enzymes, and high doses of antioxidant supplements have the potential to remove the necessary small ROS levels and thereby interfere with this induction of defenses [21,27,28].

In conclusion, a prudent course of action for improving vision and lowering the risk of eye disease (as well as a host of other diseases) at this time is to consume a whole-food-based diet rich in zeaxanthin, lutein, various phenolics, antioxidant vitamins and minerals, with a balanced ratio of omega-3 to omega-6 fatty acids, and to avoid high-caloric, high-glycemic, saturated-, *trans*-, and omega-6-fat-rich foods as well as smoking, excess alcohol, and physical inactivity. A “healthy lifestyle with a diet containing food rich in antioxidants, especially lutein and zeaxanthin, and (omega-3) fatty acids” has been recommended to prevent or slow progression of AMD and cataracts [73]. A multi-vitamin-multi-mineral supplement (each around the RDA level) with a combination of vitamins C and E, β-carotene, and zinc with copper has been recommended for AMD but not cataracts [76]. Optimal doses of supplements have yet to be determined, but high-dose xanthophyll and/or antioxidant vitamin supplements are unlikely to be beneficial (cf. also [77]).

## 6. Relating the Mechanisms of Photoprotection to the Photochemistry of Photosynthesis and Human Vision

**Figure 2:**
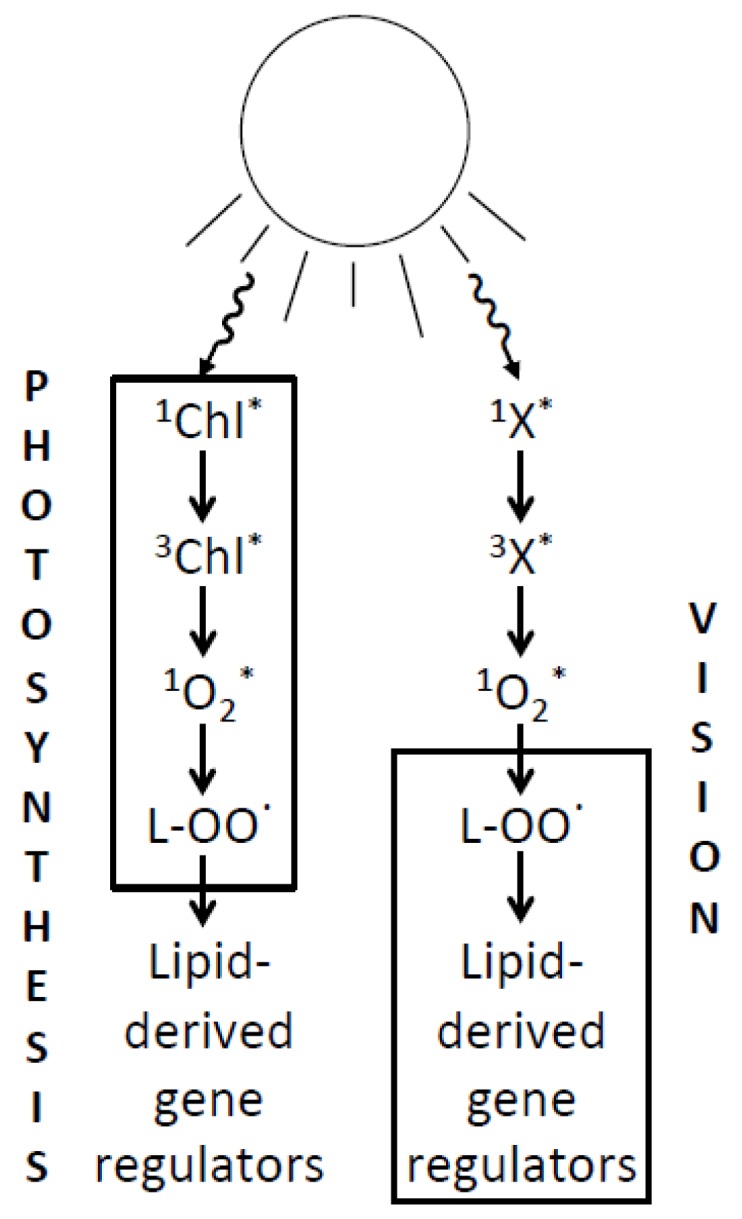
Comparison of photosynthesis and vision with respect to the principal steps in the series of reactions from light absorption, by various chromophores, to transfer of excitation energy to singlet oxygen (^1^O_2_*) resulting in lipid peroxidation and formation of lipid-derived gene regulators. Light absorption occurs in chlorophyll (Chl)-antenna-protein complexes in photosynthesis, and in retinal-opsin-complexes in vision. Triplet excited chlorophyll (^3^Chl*) is known to act as the photosensitizer in photosynthesis, passing excitation energy to oxygen, thus forming singlet oxygen. The nature of the photosensitizer in vision (X) is currently under debate. In contrast to protein-bound Chl, opsin-bound retinal does not produce singlet oxygen. However, there is current debate that, once released from opsin, all-*trans* retinal absorbs another photon and may give rise to singlet oxygen formation (see, e.g., [78]). The boxes around the different phases of the reaction series serve to indicate where research focus has previously been placed.

Continuing comparison of parallels between light absorption, light processing, and protection against excessive excitation in photosynthesis and human vision may be rewarding (Figure 2). Research on the photoprotection of photosynthesis has focused, e.g., on interaction of the light-absorbing chlorophylls with xanthophylls in the light-collecting system, with a lesser focus on lipid peroxidation-based messengers and signaling networks (Figure 2). On the other hand, research on human vision has identified synergies among factors involved in signaling networks, while the photo-physics of excited states of rhodopsin derivatives and their possible interaction with xanthophylls have not been fully elucidated (Figure 2). Possible practical applications based on insight into the photo-physics of visions will be addressed below.

The parallels between photosynthesis and human vision are remarkable—as would be expected from an evolutionary viewpoint considering not only the homology of these systems, but also common dictates from natural selection and adaptive advantage. Both systems must be able to collect photons of light (as carriers of information in vision, or carriers of energy in photosynthesis) via light-absorbing pigments or chromophores (retinal or chlorophyll), which is achieved by the chromophore’s binding to a protein (opsin or chlorophyll-binding proteins, respectively). Figure 2 summarizes some of the parallels between human vision and photosynthesis.

Chromophore-binding proteins, such as the chlorophyll-binding proteins of photosynthesis, typically help increase the lifetime of a chromophore’s excited state (produced by absorption of a photon of light) long enough to allow highly efficient transfer of excitation energy (e.g., into an electron transport chain in the case of photosynthesis). Increasing the chromophore’s lifetime, however, comes at a cost under high light levels; if the chromophore’s excited state builds up just for fractions of a second, a conversion (via intersystem crossing) occurs to a slightly lower (triplet) excited state of the chromophore able to pass on excitation energy to oxygen, thereby forming potentially highly destructive reactive oxygen species. Chromophores thus typically act as facilitators of photo-damage, or photosensitizers, although in the vision process, it is not opsin-bound retinal, but forms of retinal (all-*trans*-retinal or its derivatives) released from opsin that absorb another photon and may act as the singlet-oxygen-producing photosensitizer ([78,79,80,81,82,83,84,85,86,87]).

The accumulation of light-absorbing pigment protein complexes typically responds to long-term light availability. Plants accumulate more chlorophyll when grown in low light, and thereby become more susceptible to over-excitation and inactivation of photochemistry under high light exposure, than plants grown in high light [32]. Similarly, animals raised in low light environments accumulated more rhodopsin, and were more susceptible to high-light-induced vision loss (for an overview, see [78]). Based on these findings and the current understanding of the mechanisms of photo-damage, it is possible that long-term light environment may affect the risk for human eye disease. Future research should assess whether predominant exposure to very low light environments in typical home and office settings may increase the risk for eye disease compared to regular exposure to modest levels of natural sunlight outdoors, e.g., in mornings and afternoons or on overcast days (with typical light intensities around only 10 µmol photons m^−2^·s^−1^ for indoors fluorescent lighting versus around 300 and up to 2000 µmol photons m^−2^·s^−1^ for natural sunlight outdoors during overcast skies and full midday sunlight on clear days, respectively; B. Demmig-Adams, [79]).

It has been discussed that the human eye, just like photosynthesis, must maintain “a delicate balance between maximizing the absorption of photon for vision and retinal image quality while simultaneously minimizing the risk of photo-damage when exposed to bright light” [80]. Rhodopsin presence has been proposed to be required for photo-damage in the retina ([81]; see also [82,83]), and, as pointed out above, there has been recent discussion that all-*trans*-retinal, once released from rhodopsin during the vision cycle, or specific derivatives of all-*trans*-retinal, may accumulate in the retina and act as photosensitizer(s) (“X” in Figure 2; [78,81,82,83,84,85,86,87,88]). Lutein and zeaxanthin have been suggested to confer photoprotection against such an action [84]. It is currently unknown whether or not, or how, xanthophylls may facilitate de-excitation of any excited states of chromophores in human vision. In contrast, detailed information has been accumulated on chromophore de-excitation, and an involvement of xanthophylls, in photosynthesis (see below).

A chromophore’s first excited state is typically a singlet-excited state (e.g., ^1^Chl*; Figure 2) electronically unable to pass excitation energy to oxygen in its ground (triplet) state. However, the chromophore’s state reached after conversion via intersystem crossing is a triplet state (e.g., ^3^Chl*; Figure 2) that reacts readily with oxygen, resulting in the formation of highly reactive singlet-excited oxygen. Under high light exposure, a light-absorbing system like chlorophyll-binding proteins is thus extremely vulnerable to destruction by singlet oxygen. Multiple mechanisms have evolved to provide protection by safe de-excitation of “unwanted”, excessive excited states at every step of the above cascade, *i.e.*, (1) de-excitation of the chlorophyll’s singlet-excited state, (2) de-excitation of its triplet state, (3) de-excitation of singlet-excited oxygen, and, finally, (4) mechanisms that re-reduce oxidized lipids.

In photosynthesis, zeaxanthin (and to a lesser extent lutein) facilitate de-excitation of excessive singlet-excited chlorophyll [31,37]; efficient de-excitation of triplet-excited chlorophyll in light-harvesting complexes can also be catalyzed by lutein and zeaxanthin [89,90]. Singlet-excited oxygen (^1^O_2_*) can be de-excited by vitamin E and various carotenoids (see [91]). Remarkably, a mixture of zeaxanthin, lutein, and a lutein derivative in the concentrations found in the human retina was shown to be more effective at singlet oxygen de-excitation than each xanthophyll alone [92]. Finally, zeaxanthin can apparently directly inhibit lipid peroxidation (l-OO^•^) in plants [34,35] and may serve in a similar function in the human eye through the synergistic interaction with vitamin E described above.

The underlying photo-physical mechanism of de-excitation of singlet-excited chlorophyll facilitated by zeaxanthin has received much attention, and evidence has been provided (for reviews, see [32,93,94]) for several different mechanisms that may yet turn out to all contribute, *i.e.*, (1) direct transfer of excitation energy from singlet-excited chlorophyll to zeaxanthin (and perhaps lutein), followed by conversion of the excitation energy to harmless heat [95]; (2) reversible charge transfer between singlet-excited chlorophyll and zeaxanthin (transfer of an electron from chlorophyll to zeaxanthin and back) resulting in loss of the excitation energy as harmless heat [96]; (3) a zeaxanthin-induced change in the chlorophyll-binding light-harvesting proteins from a conformation that lengthens the lifetime of ^1^Chl* to a conformation allowing efficient return of ^1^Chl* directly to ground-state chlorophyll while releasing excitation energy as harmless heat [93,94]. Zeaxanthin’s close structural isomer, lutein, differs in the energy levels of its excited states as well as in its exact structure (and perhaps its interaction with membranes and proteins).

The evolutionary conservation of xanthophyll association with light-absorbing systems in widely different organisms is as remarkable as the multitude of mechanisms of xanthophyll-facilitated photoprotection. More research is needed to elucidate whether different organisms employ different ones of these multiple mechanisms—or whether the whole suite of mechanisms may be conserved across species and light-processing systems.
